# Inorganic phosphate nanorods are a novel fluorescent label in cell biology

**DOI:** 10.1186/1477-3155-4-11

**Published:** 2006-10-30

**Authors:** Chitta Ranjan Patra, Resham Bhattacharya, Sujata Patra, Sujit Basu, Priyabrata Mukherjee, Debabrata Mukhopadhyay

**Affiliations:** 1 Department of Biochemistry and Molecular Biology, Mayo Clinic Cancer Center, Mayo Clinic, Rochester, Minnesota, USA

## Abstract

We report the first use of inorganic fluorescent lanthanide (europium and terbium) ortho phosphate [LnPO_4_·H_2_O, Ln = Eu and Tb] nanorods as a novel fluorescent label in cell biology. These nanorods, synthesized by the microwave technique, retain their fluorescent properties after internalization into human umbilical vein endothelial cells (HUVEC), 786-O cells, or renal carcinoma cells (RCC). The cellular internalization of these nanorods and their fluorescence properties were characterized by fluorescence spectroscopy (FS), differential interference contrast (DIC) microscopy, confocal microscopy, and transmission electron microscopy (TEM). At concentrations up to 50 μg/ml, the use of [^3^H]-thymidine incorporation assays, apoptosis assays (TUNEL), and trypan blue exclusion illustrated the non-toxic nature of these nanorods, a major advantage over traditional organic dyes

## Background

Nanotechnology, the creation of new objects in nanoscale dimensions, is a cutting edge technology having important applications in modern biomedical research [1-7]. Because the dimension of nanoscale devices is similar to cellular components such as DNA and proteins [8,9], tools developed through nanotechnology may be utilized to detect or monitor several diseases at the molecular level [3,10,11]. Bio-imaging with inorganic fluorescent nanorods probes have recently attracted widespread interest in biology and medicine [1-4,12-14] compared to nanospheres. According to the reported literature [15], there is a drastic reduction of the plasmon dephasing rate in nanorods compared to small nanospheres due to a suppression of interband damping [15]. These rods show very little radiation damping due to their small volumes. These findings imply large local-field enhancement factors and relatively high light-scattering efficiencies, making metal nanorods extremely interesting for optical applications. Therefore, we are highly interested to examine the possibility of using inorganic fluorescent nanorods, especially lanthanide ortho phosphate LnPO_4_·H_2_O [Ln = Eu or Tb], as fluorescent labels in cell biology. On the otherhand, in comparison to organic dyes (including Fluorescein, Texas Red™, Lissamine Rhodamine B, and Tetramethylrhodamine) and fluorescent proteins (Green fluorescent protein, GFP), inorganic fluorescent nanoparticles have several unique optical and electronic properties including size- and composition-tunable emission from visible to infrared wavelengths, a large stokes shift, symmetric emission spectrum, large absorption coefficients across a wide spectral range, simultaneous excitation of multiple fluorescent colors, very high levels of brightness, [4,13], high resistance to photobleaching, and an exceptional resistance to photo- and chemical degradation [2-5,13,16,17] ].

Bio-conjugated inorganic nanoparticles have raised new possibilities for the ultrasensitive and multiplexed imaging of molecular targets in living cells, animal models, and possibly in human subjects. In this context, lanthanide-based inorganic fluorescents, especially Eu- and Tb-phosphate nanoparticles, have attracted a great deal of attention in cell biology. Optical properties of europium (Eu) and terbium (Tb) salts and their chelates have been used in diverse biomedical applications, namely time-resolved fluorometric assays and immunoassays [18-26]. Furthermore, there are some previous reports regarding the introduction of inorganic luminescent nanospheres such as CdSe, ZnS, PbSe, ZnSe, and ZnS into cells [4,27,28]; however, these compounds are toxic to the cells. As the potential toxic effects of nanomaterials (nanospheres or nanorods) is a topic of considerable importance, the *in vivo *toxicity of Eu and Tb salts will be a key factor in determining whether the fluorescent imaging lanthanide probes could be used *in vivo*. In our study, lanthanide phosphate [LnPO_4_·H_2_O, where Ln = Eu and Tb] nanorods were found to be non-toxic to endothelial cells as analyzed by cell proliferation assays [29] and the TUNEL assay. Moreover, to the best of our knowledge, there is no known report internalization of naked (nanorods without surface modifications of peptides, organic molecules, or polymers) fluorescent nanorods (EuPO_4_·H_2_O and TbPO_4_·H_2_O) into cells. In order to functionalize the surface of nanorods, we used aminopropyl trimethoxy silane (APTMS) or mercapto-propyl trimethoxy silane (MPTMS) as reported in the literature [30]. The functionalization of these nanorods using the microwave technique [30] is currently ongoing in our laboratory.

To the best of our knowledge, this is the first report of inorganic lanthanide phosphate fluorescent nanorods as fluorescent labels in cell biology. In the present study, EuPO_4_·H_2_O and TbPO_4_·H_2_O nanorods have been prepared by microwave heating and characterized as described previously [31]. The microwave technique is simple, fast, clean, efficient, economical, non-toxic, and eco-friendly [31]. The aim of our study was to investigate whether these inorganic fluorescent nanorods were capable of entering the cells and retaining their fluorescent properties for detection post-internalization. If so, drugs or biomolecules attached to these nanorods can then be detected after internalization and benefit future imaging, therapeutics, and diagnostic purposes. The aim of this paper is not to compare the toxicity of inorganic fluorescent nanorods with other inorganic fluorescent nanoparticles such as CdSe or CdTe but to explore and find new inorganic fluorescent materials that can be used as fluorescent labels in cell biology.

## Results and discussion

**Figure 1:**
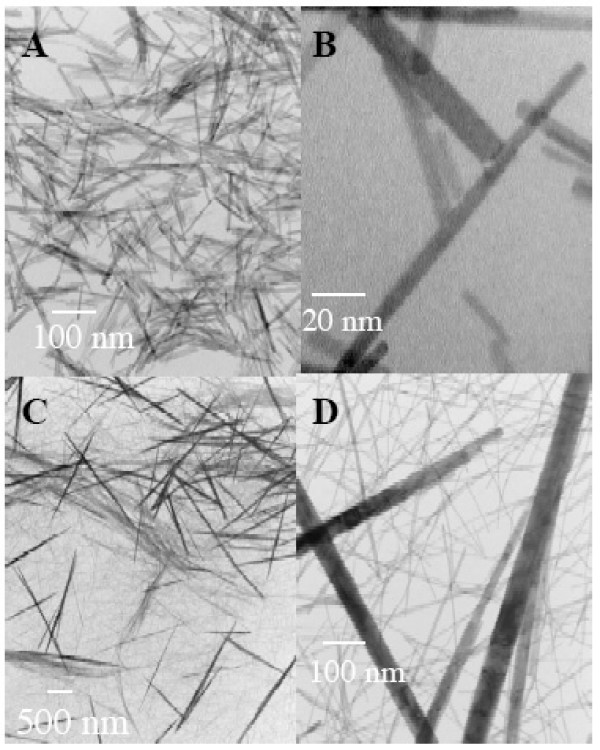
TEM images of as-synthesized (A-B) EuPO_4_·H_2_O nanorods and (C-D) TbPO_4_·H_2_O nanorods with different magnifications, respectively.

The morphologies of LnPO_4_·H_2_O [Ln = Eu and Tb] nanomaterials were further characterized by transmission electron microscopy (TEM) at different magnifications (Figure 1A–D). The TEM images of as-synthesized products clearly showed that EuPO_4_·H_2_O material (Figure 1A–B) entirely consists of nanorods [6 to 8 nm in diameter and 100 to 300 nm in length] and TbPO_4_·H_2_O products (Figure 1C–D) were a mixture of two rod types in micrometer size (small rods at 0.5 to 1.5 μm in length and 6 to 8 nm in width and bigger rods at 1.1 to 2.2 μm in length and 80 to 130 nm in width).

**Figure 2:**
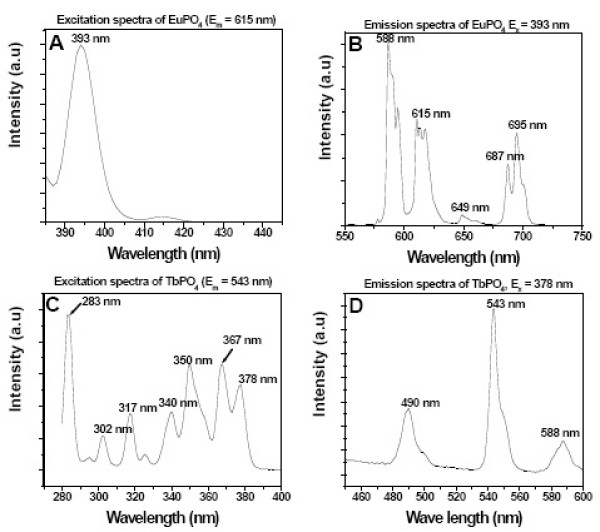
Excitation (A,C) and emission spectra (B,D) of as-synthesized EuPO_4_·H_2_O, TbPO_4_·H_2_O nanorods.

The excitation and emission spectra of LnPO_4_·H_2_O are shown in Fig. 2A–D. The main emission peaks (Fig. 2B) for EuPO_4_·H_2_O were observed at 588 nm, 615 nm, and 695 nm after excitation at 393 nm (Fig. 2A). Similarly, the main emission peaks (Fig. 2D) for TbPO_4_·H_2_O were observed at 490 nm, 543 nm (major), and 588 nm after excitation at 378 nm (Fig. 2C). The other excitation wavelengths for EuPO_4_·H_2_O were 415 nm, 444 nm, 464 nm, 488 nm (week), 525 nm, 535 nm etc (data not shown). Excitation wavelengths for TbPO_4_·H_2_O were 283 nm, 302 nm, 317 nm, 340 nm, 350 nm, 367 nm, 460 nm, 488 nm etc (all are not shown here). Excitation at any of these wavelengths resulted in similar emission spectra (data not shown) for EuPO_4_·H_2_O and TbPO_4_·H_2_O. The excitation spectrum of Eu^3+ ^(Fig. 2A) and Tb^3+ ^(Fig. 2C) revealed an intense band at 393 nm and at 283 nm (due to the f-f transitions), respectively. The emission spectrum (Fig. 2B) was composed of a ^5^D_0_-^7^F_J _(J = 1, 2, 3, 4) manifold of emission lines of Eu^3+ ^with the magnetic-dipole allowed ^5^D_0_-^7^F_1 _transition (588 nm) being the most prominent emission lines. TbPO_4_·H_2_O yielded the characteristic blue ^5^D_4_-^7^F_J' _(J' = 4,5) emission and the green ^5^D_3_-^7^F_J _(J = 3, 4,5,6) emission of Tb^3+ ^though the ^5^D_4_-^7^F_5 _(543 nm) green emission was the most prominent band (Fig. 2D). Such fluorescence properties of inorganic nanorods (LnPO_4_·H_2_O) have attracted a great deal of attention in biology because they have a strong optical emission that exhibits a sharper spectral peak than typical organic dyes, have a large Stokes shift, and are minimally influenced by other chemicals. The emission spectrum has the following salient characteristics: (i) large Stokes shift (615-393 = 222 or 543-283 = 260 dependent upon the emission wavelength of europium excitation at 393 nm or terbium excitation at 283 nm), (ii) a narrow and symmetric emission at 615 nm for europium and 543 nm for terbium, and (iii) a long-lasting existence. Therefore, our nanorods, despite its slightly larger size, satisfy all the criteria of inorganic fluorescent nanoparticles.

**Figure 3:**
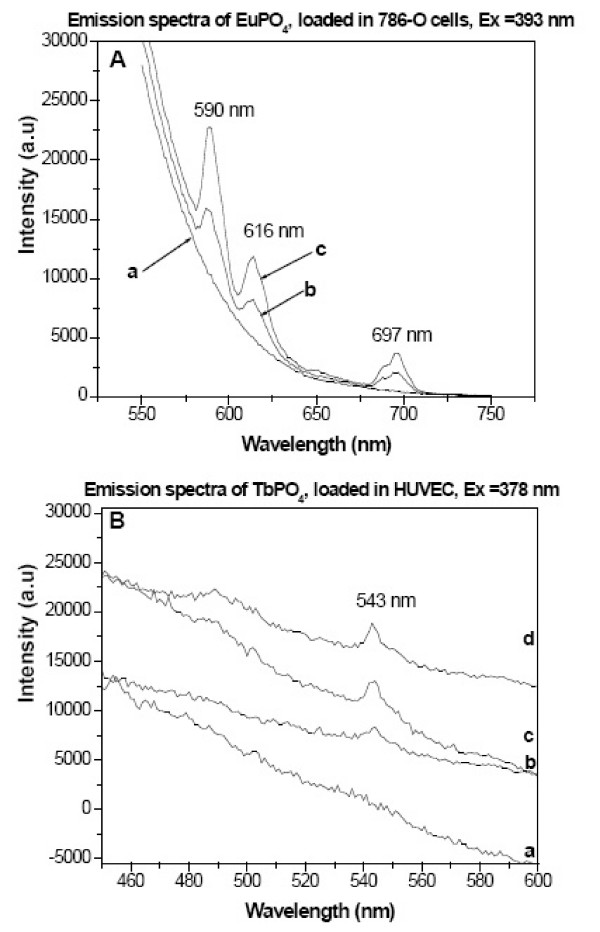
Emission spectra of (A) EuPO_4_·H_2_O nanorods loaded inside 786-O cells treated at various concentrations (a = 0 μg/ml, b = 50 μg/ml, c = 100 μg/ml), (B) TbPO_4_·H_2_O nanorods loaded inside HUVEC cells treated at various concentrations (a = 0 μg/ml, b = 20 μg/ml, c = 50 μg/ml, d = 100 μg/ml).

In order to determine if the fluorescence activity of these LnPO_4_·H_2_O nanorods remain unchanged inside the cell, 786-O cells and HUVEC are incubated for 24 hours with these nanorods at various concentrations and the emission (fluorescence) spectra were recorded on a Fluorolog-3 Spectrofluorometer after extensive washing with PBS (phosphate buffer saline) and shown in Figure 3A–B. Figure 3A shows the emission spectra of 786-O cells loaded with EuPO_4_·H_2_O nanorods at different concentrations: 0 μg/ml (curve-a), 50 μg/ml (curve-b), and 100 μg/ml (curve-c), respectively. Similarly, Figure 3B shows the emission spectra of HUVEC cells loaded with TbPO_4_·H_2_O nanorods at different concentrations: 0 μg/ml (curve-a), 20 μg/ml (curve-b), 50 μg/ml (curve-c), and 100 μg/ml (curve-d), respectively. Similar results were obtained when 786-O cells were treated with TbPO_4_·H_2_O and HUVEC cells were treated with EuPO_4_·H_2_O nanorods (data not shown). It was observed that with increasing concentrations of LnPO_4_·H_2_O nanorods (0 to 100 μg/ml), the rate of nanorod accumulation inside the 786-O and HUVEC cells increased as the fluorescence intensity from curve -a to curve -c/d increased (Figure 3A–B). As these nanorods show their distinct fluorescence properties inside the HUVEC and 786-O cells, it indirectly proves that these nanorods are internalized (which is confirmed by TEM, as discussed later).

**Figure 4:**
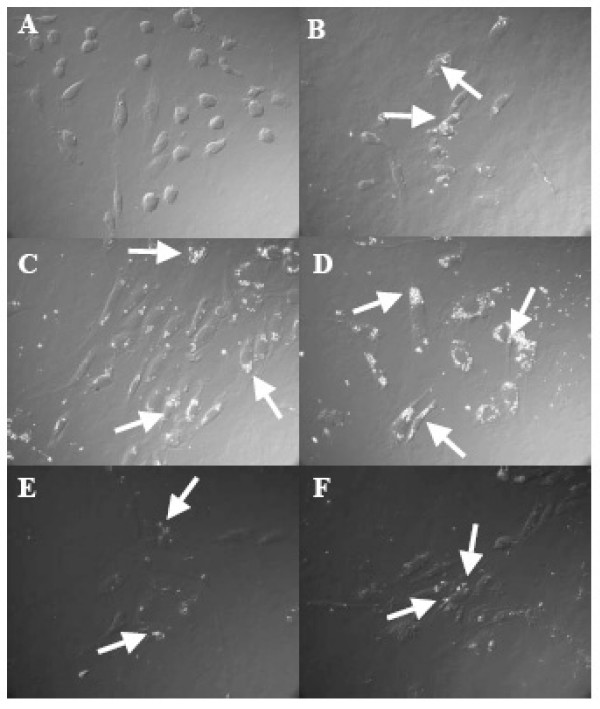
DIC microscopy pictures of HUVEC with nanorods and without nanorods. A: control HUVEC with no treatment, no nanorods were observed, (B-D): HUVEC treated with EuPO_4_·H_2_O at different concentrations (B: 20 μg/ml, C: 50 μg/ml and D: 100 μg/ml), and (E-F): HUVEC treated with TbPO_4_·H_2_O nanorods at different concentrations (E: 50 μg/ml and F: 100 μg/ml). In few places, nanorods, inside the cells, were marked by white arrow sign (B-D).

A number of methods such as differential interference contrast (DIC) microscopy, confocal microscopy and transmission electron microscopy (TEM) has been used to determine cellular trajectories of nanorods and are described below. Differential interference contrast (DIC) microscopy pictures of HUVEC (Fig. 4A–F) clearly show a significant difference in contrast between the untreated control cells (Fig. 4A), the cells treated with EuPO_4_·H_2_O (Fig. 4B–D), and the cells treated with TbPO_4_·H_2_O nanorods (Fig. 4E–F) at various concentrations. Similar results were obtained when 7886-O cells were treated with LnPO_4_·H_2_O nanorods (data not shown). These results again indirectly prove that these LnPO_4_·H_2_O nanorods are internalized.

**Figure 5:**
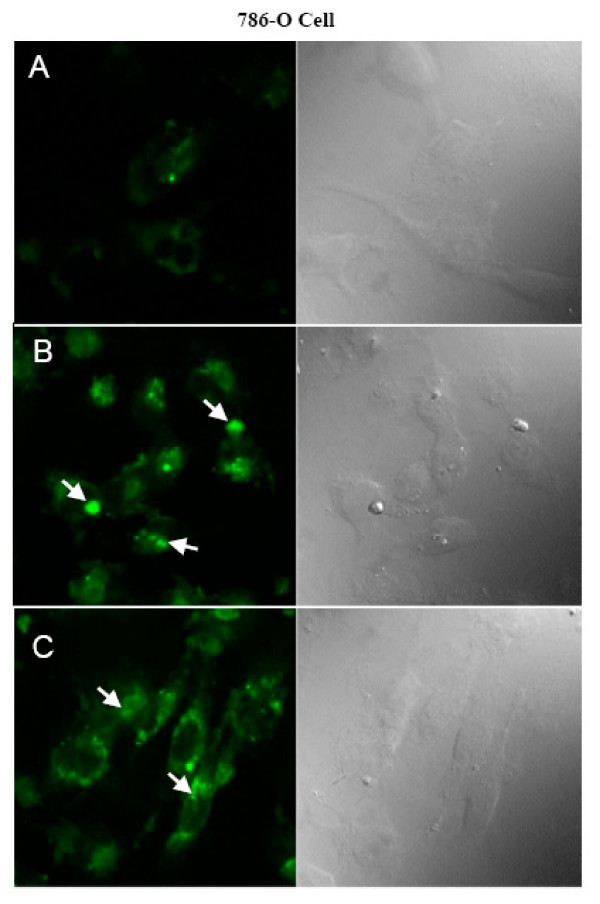
Fluoresence (First column) and their corresponding phase images (Second column) of 786-O cells treated with LnPO_4_·H_2_O nanorods. (A): Control 786-O cells with no treatment, slight green color due to auto fluorescence in (A), (B): 786-O cells treated with EuPO_4_·H_2_O nanorods, and (C): 786-O cells treated with TbPO_4_·H_2_O nanorods, taken by confocal microscope. In few places green fluorescence color of nanorods inside the cells, were marked by white arrow sign.

Inorganic fluorescent EuPO_4_·H_2_O and TbPO_4_·H_2_O nanorods inside the 786-O cells (Fig. 5) and HUVEC (data not shown here) were detected by confocal microscopy. The fluorescence (left column) and their corresponding phase images of untreated control cells (Fig. 5A), cells treated with EuPO_4_·H_2_O nanorods (Fig. 5B), and cells treated with TbPO_4_·H_2_O nanorods (Fig. 5C) were shown. The EuPO_4_·H_2_O nanorods have a useful excitation region from 250 to 535 nm with a maximum at 393 nm [26]. In this study, confocal fluorescence microscopy images and phase images of cells were collected through the use of a Zeiss LSM 510 confocal laser scan microscope with a C-Apochromat 63 X/NA 1.2 water-immersion lens in conjunction with an Argon ion laser (488 nm excitation). The fluorescence emission was collected with a 100X microscope objective then spectrally filtered using a 515 nm long pass filter. Analysis by confocal laser scanning microscopy (excitation at λ = 488 nm) shows the presence of green fluorescent structures scattered in the cytoplasmic compartments of cells treated with nanorods (Fig. 5B–C). It was also observed that there were very few green fluorophores (Fig. 5A) inside the cells due to auto-fluorescence whereas in Fig. 5(B–C), fluorophores were clearly observed due to the presence of Eu^3+ ^and Tb^3+ ^ions in crystallized LnPO_4_·H_2_O nanorods. Overall, there is a significant difference in fluorescence between untreated control cells (Fig. 5A) and nanorods treated cells (Fig. 5B–C). These results prove the internalization of LnPO_4_·H_2_O nanorods inside 786-O cells. Similar results were obtained when HUVEC were treated with LnPO_4_·H_2_O nanorods (data not shown). On the otherhand, a red emission was expected from cells treated with EuPO_4_·H_2_O nanorods. Unfortunately, we could not distinguish the huge fluorescence intensity between untreated control cells and nanorod-treated cells when we collected the emission spectra in red region. Therefore, we have collected the emission spectra for EuPO_4_·H_2_O- loaded cells in the green emission region (515 nm long pass filter). However, the confocal experiments for best fluorescence images are currently under detailed investigations in our laboratory.

Excitation and emission spectra of EuPO_4_·H_2_O and TbPO_4_·H_2_O nanorods were detected at the recommended wavelength by a spectrofluorometer, indicating that properties of the nanorods remained unchanged upon internalization into cells (Fig. 3A–B). However, for confocal microscopy, the same recommended excitation wavelengths were not available on the instrument. Thus, we took confocal images after excitation at 488 nm and collected emission with a 515 nm long pass filter. We found that after excitation at 488 nm and collected the emission spectrum with a 515 nm long pass filter, there was a significant and clear distinction between the fluorescence intensity of untreated cells (Fig. 5A) and nanorod-treated cells (Fig. 5-C). However, after scanning through a number of different excitation wavelengths as reported in the literature [26], we could not clearly distinguish between the fluorescence intensity of untreated cells and nanorod-treated cells. Because this is our first report using inorganic lanthanide phosphates (EuPO_4_·H_2_O and TbPO_4_·H_2_O) as a fluorescent biological label, there is no evidence to show that an emission is detectable with a 515 nm long pass filter. However, it was reported in the literature that a 488 nm excitation wavelength [26] was used in confocal microscopy to detect luminescent properties of europium (III) nanoparticles.

**Figure 6:**
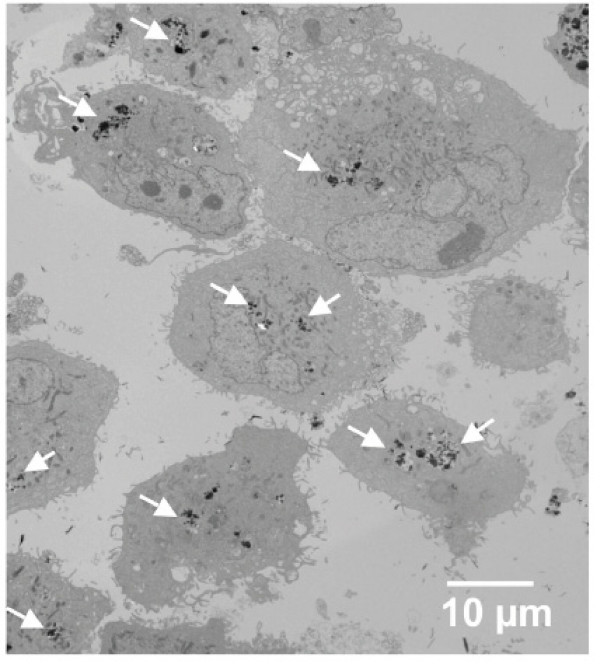
EuPO_4_·H_2_O fluorescent nanorods, were visualized by TEM inside the cytopplasmic compartments of 786-O cells. In few places, EuPO_4_·H_2_O nanorods, inside the cells, are marked by white arrow signs.

**Figure 7:**
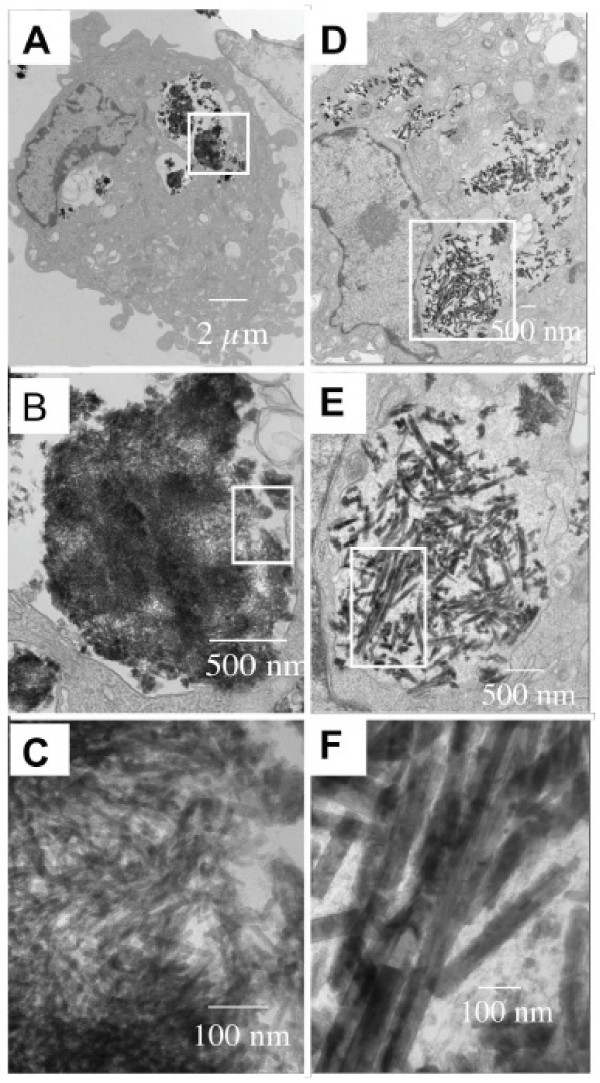
Fluorescent LnPO_4_·H_2_O nanorods were visualized by TEM inside the cytoplasmic compartments of HUVEC. (A-C) EuPO_4_·H_2_O nanorods and (D-F) TbPO_4_·H_2_O nanorodsare observed inside the HUVEC with increasing magnifications. B was the enlarge picture of white block in A, C was the enlarge picture of white block in B. Similarly, E was the enlarge picture of white block in D and F was the enlarge picture of white block in E.

The TEM image of 786-O cells treated with EuPO_4_·H_2_O nanorods was shown in Fig. 6. This figure clearly indicated that in most of the cells, uptake of these nanorods occurred. Fig. 7A–C and Fig. 7D–F represent the TEM images of HUVEC cells treated with EuPO_4_·H_2_O nanorods and with TbPO_4_·H_2_O nanorods, respectively, illustrating that both nanorods could enter the cytoplasmic compartments. The morphology of these cells also clearly demonstrated that they were healthy after internalizing these materials (Fig. 6 and Fig. 7) though their spherical shape was due to trypsinization, neutralization with TNS, and fixation in Trumps solution for TEM. Similarly, the morphology of the fluorescent nanorods remained unchanged after internalization. Similar results were obtained when the 786-O cells were treated with LnPO_4_·H_2_O nanorods (data not shown). From the combination of Fig. 1D and Fig. 7F, it appears that the small rods seen in Figure 1D were not internalized by the endothelial cells as illustrated with TEM (Fig. 7F). However, other than the larger TbPO_4_·H_2_O nanorods, some aggregated rods were visible in the cytoplasm. It is possible that these smaller rods aggregate similar to cadmium-based salts [32] but are notably less toxic when taken up by endothelial cells.

Considering our results from fluorescence spectroscopy, DIC, confocal, and TEM, we've shown that these fluorescent nanorods can be internalized in a cellular system and are readily visualized by microscopy. These nanorods then offer a useful alternative as fluorescent probes for targeting various molecules to specific cells. The exact mechanism for internalization of these nanorods still remains unclear but is under investigation in our laboratory.

**Figure 8:**
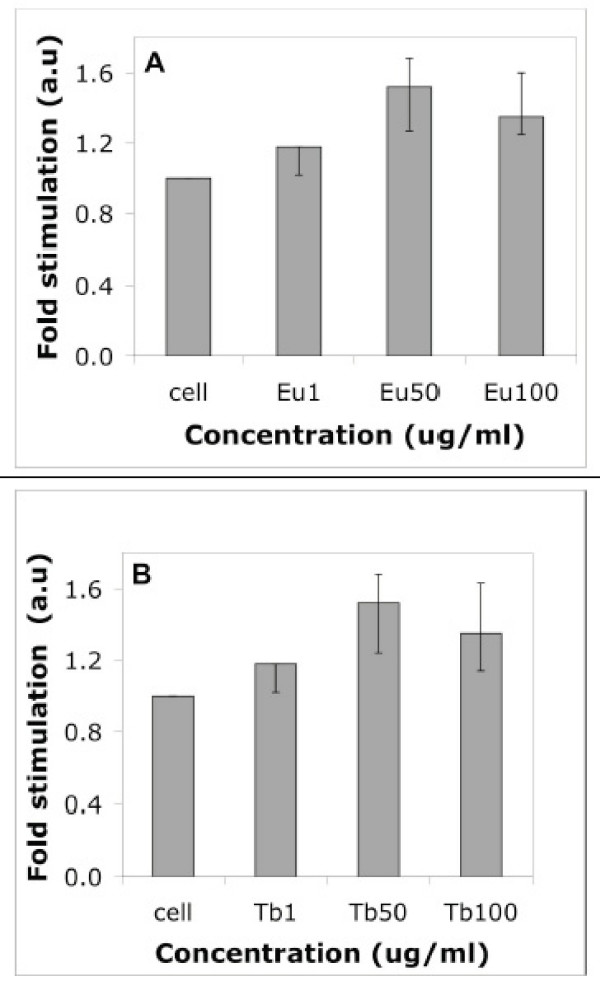
Effect of fluorescent nanorods (EuPO_4 _and TbPO_4_) with different concentrations to normal HUVEC was observed by [^3^H]thymidine incorporation asssay. A serum-starved HUVEC was treated with (A) EuPO_4_·H_2_O nanorods and (B) TbPO_4_·H_2_O nanorods at the concentration range of 1–100 μg/mL [Eu1 = 1 μg/ml, Eu50 = 50 μg/ml, Eu100 = 100 μg/ml. Similarly, Tb1 = 1 μg/ml, Tb50 = 50 μg/ml, Tb100 = 100 μg/ml]. Average of three independent experiments, each was done in triplicate.

Since these inorganic nanorods show distinct fluorescence activity upon cellular internalization, we have decided to use these materials as a fluorescent label for HUVEC and 786-O cells. We examined their *in vitro *toxicity with [^3^H]-thymidine incorporation assays [29] on normal endothelial cells (HUVEC) and found them to be non-toxic (Fig. 8A–B). Although there were indications that exposure to certain nanomaterials might lead to adverse biological effects, this appears to dependent upon the chemical and physical properties of the material [4,27,28]. The potential toxicity of inorganic fluorescent nanoparticles has recently become a topic of considerable importance and discussion, especially since *in vivo *toxicity is likely to be a key factor in determining whether fluorescent probes will be approved by regulatory agencies for human clinical use. HUVEC proliferation [29] was clearly not affected from internalization of materials up to 50 mg/ml compared to control samples (Fig. 8A–B); however, at concentrations greater than 50 mg/ml, nanorods were detected to be toxic. Experiments were repeated in triplicate and results were reproducible.

To observe viability, HUVEC were treated with 50 μg/ml of europium and terbium phosphate nanorods for 24–48 hours. There was no difference in cell death between untreated control cells (no treatment) and nanorod-treated cells as assessed by trypan blue (data not shown). These results illustrate a biocompatibility between the nanorods and the cells.

**Figure 9:**
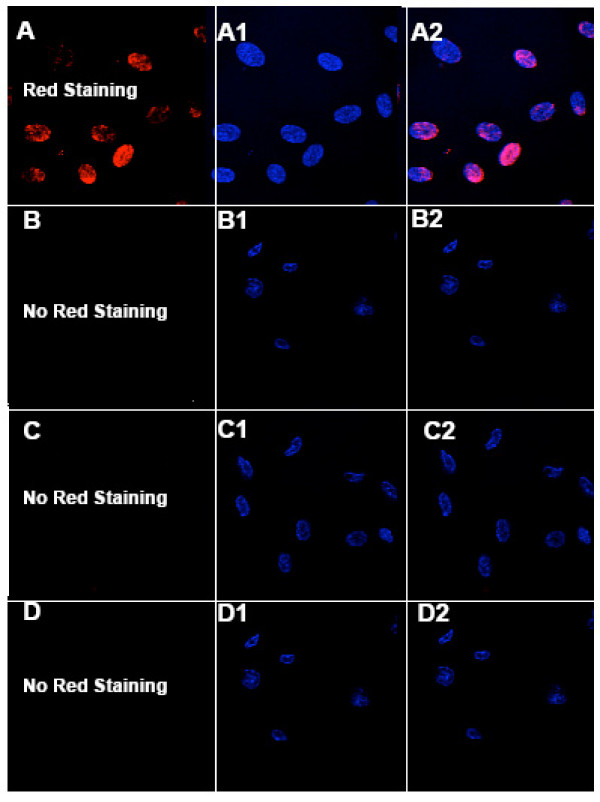
TUNEL assay apoptosis of HUVEC. First row: positive control experiment, second row: untreated control experiment, third row: HUVEC treated with EuPO_4_·H_2_O at 50 μg/ml for 20 h of incubation at 37°C and fourth row: HUVEC treated with TbPO_4_·H_2_O at 50 μg/ml for 24 h of incubation at 37°C. TUNEL assay apoptosis of HUVEC using camptothecin (4 h incubation at 37°C) as positive inducer (First row). A: TMR red -stained nuclei of HUVEc appear in red color due to presence of apoptotic cells, A1: The DAPI-stained nuclei appear in blue and A2: merged picture of A and A1. First Column: The nuclei of HUVEC were stained with TMR red (B-D), red staining was not observed due to absence of no apoptotic cells. Second column: The DAPI-stained nuclei appear in blue (B1-D1), and Third column: merged picture of first and second column (B2-D2).

To investigate whether uptake of these nanorods induce apoptosis, we assayed endothelial cells treated with LnPO4.H2O nanorods using two apoptotic methods: (i) fluorescence microscopy using the In Situ Cell Death Detection Kit, TMR red (Roche, Cat. No.#12 156 792 910) and (ii) flow cytometry using Annexin V-FITC Apoptosis Detection Kit (Biovision, Cat. No. K101-100). The TUNEL assay detects apoptosis-induced DNA fragmentation through a quantitative fluorescence assay and was performed according to the manufacturer's instructions. In tunnel assay, the positive control apoptosis has been induced in cells using camptothecin (~2.5 mM) for 4 h of incubation (Fig. 9(A-A2)). The red-colored (TMR red-stained nuclei) apoptotic cells (Fig. 9A) were visualized under a microscope, counted (6 fields per sample), and photographed using a digital fluorescence camera. The DAPI-stained nuclei appeared blue in Fig. 9.A1 and Fig. 9.A2 shows the merged images of TMR- and DAPI-stained cells. The results of the TUNEL assay for the untreated control HUVEC and HUVEC cells treated with LnPO_4_·H_2_O nanorods are shown in Fig. 9B–D. In the first column (B-D) of Figure 9, no nuclei of TMR red-stained HUVEC cells were detected due to the absence of apoptotic cells. Blue DAPI-stained nuclei are in the second column (B1-D1) and the third column (B2-D2) shows the merged images. There was no difference in the number of apoptotic cells (~0%) detected in the untreated control experiment (First row: B, B1 and B2) nor cells treated with EuPO_4_·H_2_O nanorods (second row: C, C1 and C2) and TbPO_4_·H_2_O nanorods (third row: D, D1 and D2). The results of Fig. 6 and Fig. 9 clearly indicate that these nanorods were not toxic to endothelial cells. Similarly, flow cytometry analysis yielded no difference in the number of apoptotic cells bewteen untreated controls and nanoparticle-treated (data not shown).

Parak *et al*. [32] has indicated that the cellular toxicity of stable nanomaterials is primarily due to aggregation rather than the release of Cd elements. However, in our case, since these nanorods are based on an entirely different material than cadmium, their mechanism is likely to be different than Cd-based materials. Therefore, if the toxicity of Cd-based materials is due to an aggregation of ion, that may not be the case for nanorods as supported by our data.

While there is no direct evidence for the effect of nanoparticle size on internalization and toxicity, some reports indicate that nanoparticle size is involved [28,32,33]. In our case, we are currently studying in detail the cytotoxicity and mechanism for the cellular internalization of these nanorods. Finally, we should mention in our experiments, the correct control would be a non-fluorescent lanthanide phosphate compound instead of untreated cells. We are currently working on the synthesis of such a reagent. Along with this work, we are also determining: (a) the mechanism of internalization; (b) the cytotoxicity of these materials; (c) the photostability and quantum efficiency of these materials; (d) the surface functionalization of these materials; (e) drug delivery using these nanorods after surface modifications; and (f) the comparison between the fluorescent and non-fluorescent lanthanide phosphate compounds in all experiments.

Nanorods are stable at room temperature indefinitely. We have performed chemical characterizations (XRD, TGA, DSC, TEM, fluorescence properties) on samples that are 4–5 months old and have detected no difference between freshly prepared nanorods and older samples including the absence of any agglomeration.

## Conclusion

A novel alternative to conventional organic dyes, we have reported the use of inorganic fluorescent EuPO_4_·H_2_O and TbPO_4_·H_2_O as a fluorescent label in biomedical research. We have shown internalization of EuPO_4_·H_2_O and EuPO_4_·H_2_O nanorods by both 786-O cells and HUVEC using fluorescence spectroscopy (FS), DIC, confocal microscopy, and TEM. The nanorods were observed to localize mainly in the cytoplasmic compartments of cells and did not appear to detrimentally affect cell viability nor induce any toxicity after internalization. These unique fluorescent nanorods offer new advancements in the detection and diagnosis for cancer therapy at an early stage and we are currently working on functionalizing these nanorods as well as utilizing them as specific vehicles for drug delivery.

## Experimental procedures

### Materials

Europium (III) nitrate hydrate [Eu(NO_3_)_3_·xH_2_O, 99.99%], terbium (III) nitrate hexahydrate [Tb(NO_3_)_3_·6H_2_O, 99.999%], ammonium dihydrogenphosphate, [NH_4_H_2_PO_4 _99.999%], were purchased from Aldrich, USA. [^3^H]-Thymidine was purchased from Amersham Biosciences, Piscataway, NJ. 786-O cells were purchased from American Type Culture Collection (ATCC, TIB-186, Rockville, MD). Dulbeco's Modification of Eagle's Medium (DMEM, 1X) was purchased from Cellgro, Mediatech, Inc, Herndon, VA, USA. Endothelial Cell Basal Medium (EBM), human umbilical vein endothelial cells (HUVEC) were obtained from Cambrex Bio Science alkersvile, Inc, MD, USA.

### Microwave-assisted synthesis of lanthanide ortho phosphate hydrates (LnPO_4_·H_2_O)

The inorganic fluorescent nanoparticles (LnPO_4_·H_2_O) were synthesized using microwave techniques as reported in the literature [31]. In a typical synthesis, 20 ml 0.05(M) of aqueous NH_4_H_2_PO_4 _were added to 20 ml 0.05 (M) of an aqueous solution of Ln(NO_3_)_3 _(Ln = Eu and Tb) in a 100 ml round-bottomed flask. The pH of the solution before and after the reaction was in the range of 1.8 – 2.2. The sample was irradiated for 20 min with 50% of the instrument's power. The microwave refluxing apparatus was a modified domestic microwave oven (GOLD STARR 1000W with a 2.45 GHz), described previously [34]. In the post-reaction treatment, the resulting products were collected, centrifuged at 36303 g (20,000 rpm with r_av _= 8.125 cm), washed several times using ethanol and distilled water, and then dried overnight under vacuum at room temperature. The yield of the as-prepared products is more than 95%.

### Cell culture experiments

HUVEC and 786-O cells were cultured at 10^5 ^cells/2 ml in six well plates for ~24 h at 37°C and 5% CO_2 _in EBM and DMEM complete media. For investigating the cellular localization (using confocal microscope), cells were plated on glass cover slips and grown to 90% confluence, and then incubated with LnPO_4_·H_2_O nanorods at a concentration of 50–100 μg/ml. After 20 h of incubation, the cover slips were rinsed extensively with phosphate buffered saline (PBS) and cells were fixed with freshly prepared 4% paraformaldehyde in PBS for 15 min at room temperature and then re-hydrated with PBS. Once all the cells were fixed, the cover slips were mounted onto glass slides with Fluor Save mounting media and examined with DIC and confocal microscopy. For detection of apoptosis using the TUNEL assay (Roche, USA, Cat. No. # 12 156 792 910), cells were mounted onto glass slides with mounting media containing DAPI (4'-6-Diamidino-2-phenylindole).

In another set of experiments, 786-O and HUVEC cells (10^5 ^cells/2 ml) were cultured in six well plates and treated with LnPO_4_·H_2_O nanorods in corresponding DMEM and EBM complete media without cover slips. After 20 h of incubation with the nanorods, the cells were washed with PBS, trypsinized, and neutralized. The cells were washed by centrifugation and re-suspended in PBS and analyzed with fluorescence spectroscopy, TEM, and flow cytometry (for detection of apoptosis of cells using annexin-FTIC-PI, Bio Vision, USA, catalog # K101-100). Cell viability for another set of cells was determined through staining with trypan blue and cells were counted using a hemocytometer.

### Cell proliferation assay

Cell proliferation to measure *in vitro *toxicity was performed with the [^3^H]-thymidine incorporation assay according to the reported literature [29]. Briefly, endothelial cells (HUVEC; 2 × 10^4^) were seeded in 24-well plates, cultured for 2 days in EBM, serum-starved (0.1% serum) for 24 hours, and then treated with different concentrations (1–100 μg/mL) of LnPO_4_·H_2_O (Ln = Eu, Tb). After 20 hours, 1 μCi [^3^H] thymidine was added to each well. Four hours later, cells were washed with cold PBS, fixed with 100% cold methanol, and collected for the measurement of trichloroacetic acid-precipitable radioactivity [29]. Experiments were repeated in triplicate and all results were reproducible.

### Apoptosis assay

Cells were seeded into 6-well plates at a density of 2 × 10^5 ^/2 ml of medium per well and grown overnight. After appropriate treatment with these nanorods (50 μg/mL), cells were extensively washed with PBS and tested with the Annexin V-FITC Apoptosis Detection Kit (Biovision, Cat. No. #K101-100) per the manufacturer's instructions. In addition, apoptosis was also determined by the TUNEL assay using the In Situ Cell Death Detection Kit, TMR red (Roche, Cat. No. #12 156 792 910). The red apoptotic cells were visualized on a microscope, counted (6 fields per sample), and photographed using a digital fluoresence camera.

## Characterization techniques

### Transmission electron microscopy study

Particle morphology (microstructures of the samples) was studied with TEM on a FEI Technai 12 operating at 80 KV. To visualize the internalization of particles inside the cells, we have folllowed the published literature procedures [35,36].

### Fluorescence microscopy

The excitation and emission (fluorescence) spectra were recorded on a Fluorolog-3 Spectrofluorometer (HORIBA JOBINYVON, Longjumeau, France) equipped with a xenon lamp as the monochromator excitation source.

### Differential interference contrast microscopy (DIC)

After fixation of cells on cover slips, the cells were mounted onto glass slides with Fluor Save mounting media and examined for DIC. Pictures were captured with AXIOCAM high-resolution digital camera using an AXIOVERT 135 TV microscope (ZEISS, Germany).

### Confocal fluorescence microscopy

Two dimensional confocal fluorescence microscopy images were collected through use of LSM 510 confocal laser scan microscope (Carl Zeiss, Inc., Oberkochcn, Germany) with C-Apochromat 63 X/NA 1.2 water-immersion lense, in conjunction with an Argon ion laser (488 nm excitation). The fluorescence emissions were collected through a 515 nm long pass filter.

After mounting the cells onto glass slides with DAPI, images were collected through a LSM 510 confocal laser scan microscope (Carl Zeiss, Inc., Oberkochcn, Germany) with a C-Apochromat 63 X/1.2 na water-immersion lens. The fluorescence emissions were collected through a 385–470 nm band pass filter in conjunction with an argon ion laser excitation of 364 nm for DAPI. The fluorescence emissions were collected through a 560–615 nm band pass filter in conjunction with a HeNe1 ion laser excitation of 543 nm for TMR red.

## Authors' contributions

CRP conceived the study and did the experiments and data analysis. SP coordinated some cell culture experiments. RB, SB, and PM also conceived the study and participated in its design and coordination and helped to draft the manuscript. DM provided guidance with the experimental design and manuscript preparation. All authors read and approved the final manuscript.
